# Assessment of Canine Autologous Conditioned Plasma^TM^ Cellular and Transforming Growth Factor-β1 Content

**DOI:** 10.3389/fvets.2018.00105

**Published:** 2018-06-11

**Authors:** Samuel P. Franklin, Kate E. Birdwhistell

**Affiliations:** ^1^Department of Small Animal Medicine and Surgery, University of Georgia, Athens, GA, United States; ^2^Regenerative Bioscience Center, University of Georgia, Athens, GA, United States

**Keywords:** canine, platelet-rich plasma, ACP, PRP, platelet activation, growth factors

## Abstract

To evaluate (1) the cellular composition of canine ACP™ including using two different preparation protocols with variations on centrifugation time, (2) the effect of different activation protocols on the transforming growth factor (TGF)-β1 content in the ACP, and (3) patient factors that might influence platelet concentration of the ACP.

ACP was made with blood from 15 dogs using a manufacturer-recommended protocol^[1]^. Each ACP sample was divided into three aliquots that were activated with calcium chloride (CaCl_2_), human γ-thrombin (HGT), or not activated. TGF-β1 was quantified in each aliquot using an ELISA and comparisons among activation protocols were performed using a Skillings-Mack test. Correlations between platelet and TGF-β1 concentration were assessed with a Pearson correlation coefficient. ACP was subsequently prepared from an additional 17 dogs using a slightly modified centrifugation protocol and cellular composition was assessed. Effects of dog age, body weight, and hematocrit were assessed for their potential impact on ACP platelet concentration using a multiple linear regression analysis.

The mean increase in platelet concentration in the ACP above that in the whole blood was 1.2× (±std 0.62) and leukocyte concentration was a mean of 26% (0.37) that in the whole blood using the standard protocol. There was a significant (*p* < 0.01) effect of activation on TGF-β1 concentrations with mean concentrations of 4,538 (2,317), 14,948 (13,784), and 14,096 (15,210) pg/ml in aliquots that were not activated or were activated with thrombin or CaCl_2_ respectively. There were significant correlations between the platelet concentration and TGF-β1 concentration in aliquots that were activated with either thrombin (r = 0.66; *p* < 0.01) or CaCl_2_ (r = 0.86, *p* < 0.0001). The mean increase in platelet concentration was 1.4× (0.62) and the leukocyte concentration was 0.28× (0.13) that in whole blood using the modified ACP preparation protocol. Dog age, body weight, and hematocrit were not significant predictors of ACP platelet concentration.

These data show that on average this preparation protocol produces a mildly platelet-concentrated, leukoreduced platelet-rich plasma. Intentional activation had a significant effect on TGF-β1 concentrations with use of both CaCl_2_ and thrombin resulting in higher TGF-β1 concentrations than that obtained in samples that are not activated.

## Introduction

Platelet-rich plasma (PRP) is being studied and used clinically with increasing frequency in canine veterinary medicine (1–22). However, several studies have documented that, as with human PRPs, the cellular composition of canine PRPs vary depending upon the PRP preparation system used (5, 7, 23). Therefore, conclusions about the efficacy of PRP in general are tenuous and it has been advised that conclusions about efficacy should ideally be made with reference to specific PRP preparations that are well characterized (7).

Canine autologous conditioned plasma (ACP^a^) has been used in numerous studies investigating both its *in vivo* efficacy as well as its cellular composition (1–3, 5, 16). Although all studies in dogs have reported benefit, the cellular composition of canine ACP has varied among the different studies that have reported its platelet and leukocyte composition (1–3, 5, 16). The first study of canine ACP reported that the platelet concentration in the ACP was approximately equivalent to that in whole blood (16). Two more recent studies evaluating the efficacy of canine ACP in research dogs specified that the mean platelet concentration was 2.4 and 2.5 times that in the whole blood (2, 3). Conversely, yet another study evaluated the cellular composition of ACP and reported that the platelet concentration was decreased by 91% in comparison to the whole blood platelet concentration (5). There were numerous differences among these studies including whether anti-coagulant was used, who performed the study and therefore who was manually isolating the ACP, and how the cellular composition was assessed, among other possible differences (2, 3, 5, 16). As it currently stands, the explanations for the great discrepancies among these studies remains unclear and clarification as to the cellular composition of ACP is needed.

Just as the cellular content of canine ACP is not clear there is also a paucity of information on growth factor content in ACP. One of the aforementioned studies quantified numerous growth factors in the ACP but the ELISAs were not validated for use with canine citrated plasma (16). In addition, while the data showed that the mean TGF-β1 concentration was at least 2,000 pg/ml, the exact concentration of TGF-β1 in the samples could not be specified because the ELISA had an upper limit of quantification of 2,000 pg/ml (16). Given that TGF-β1 concentrations in activated canine PRP samples have been reported to be as high as 70,000 pg/ml, a more well-defined understanding of the growth factor delivery with ACP would be ideal (23). Such assessment would ideally include evaluation of ACP samples that have been intentionally activated with thrombin since previous work in dogs showed that the greatest platelet activation and growth factor release occurred with thrombin activation (23). The previous study assessing growth factor concentrations in ACP evaluated samples that had not been intentionally activated, although the samples had been frozen and thawed, a process that has been shown to increase TGF-β1 concentration above that in samples that are not activated (16, 23).

The objectives of this study included performing an evaluation of the cellular composition of canine ACP. A second objective of the study was to evaluate the TGF-β1 content of ACP including in samples that had been intentionally activated with either calcium chloride or thrombin. Furthermore, after completing the first portion of this study, the objective of the second part of the study was to determine whether decreasing the centrifugation time of any necessary second centrifugation cyclewould result in an increase in mean platelet concentration in the ACP. Lastly, we sought to evaluate whether there were any predictive variables, such as dog size, age, or hematocrit, that influenced the platelet concentration in the ACP.

## Methods

### Animals

This study was approved by the Clinical Research Committee at the University of Georgia. Blood was collected from 15 healthy dogs owned by students and staff as part of this study all of whom provided written consent for inclusion. All dogs included were between 1 and 10 years of age, a minimum of 15 kg, had no medical problems or medical history of note except for possibly osteoarthritis, and were not allowed to be taking any medications other than nutritional or nutraceutical supplements such as glucosamine and chondroitin.

### Blood Collection and ACP Preparation

Each dog was sedated with 5 micrograms/kg of dexmedetomidine^[2]^ and 0.5 mg/kg of nalbuphine^[3]^ given intravenously. The dogs were placed in lateral recumbency and an area over the jugular vein was clipped of hair and aseptically prepared. An 18-gauge 2” intravenous catheter was placed. Two ACP syringes, each preloaded with 1.5 mls of ACD-A^[4]^, were sequentially filled to a total volume of 15mls. The syringes were immediately inverted to mix the blood and anti-coagulant and then placed on a blood tube rocker for continued mixing until ACP preparation. An additional 0.5 mls of blood was collected into a 1 ml syringe and then placed in a small blood tube with EDTA for complete blood count analysis of a baseline whole blood sample. All complete blood counts were performed using the same hemacytometer^[5]^.

The syringes were then spun in a centrifuge with a four-bucket swing out rotor^[6]^ at 1500 RPM for 5 min with the break disengaged. We aimed to collect 2 mls of ACP per syringe while collecting either zero or only a trace amount of blood. Consequently, if the whole blood did not separate sufficiently to enable acquisition of the desired 2mls of ACP per syringe, which generally required that there be at least 3 mls of plasma above the packed red blood cell layer, the syringes were centrifuged at 1500 RPM for an additional 10 min. One exception to this protocol was that one blood sample was spun 3 times for 5 min at 1500 RPM. The ACP from each of the two ACP syringes were collected and combined into one 15 ml conical vial, mixed, and then subsequently divided into aliquots for activation and assessment of TGF-β1 concentration.

### ACP Activation

The ACP was divided into 4 aliquots. One aliquot (400 microliters) was placed in a small EDTA tube for a complete blood count and cellular evaluation. The remaining three aliquots were each 800 microliters in volume and were either not activated or were activated with CaCl_2_^[7]^ (final concentration of 20 mM) or human gamma thrombin^[8]^ (HGT; final concentration of 20 nM) as has been done in previous studies (10, 23). The activated aliquots were allowed to incubate at room temperature for 30 min. Any samples that formed a clot (CaCl_2_ only) were briefly (5–10 s) vortexed to break up the clot. All three aliquots were then centrifuged at 3,000 × *g* for 3 min to pellet any cellular contents. The supernatants were collected, placed in new tubes, and then frozen at −80°C for future TGF-β1 analysis using an ELISA.

### TGF-β1 Analysis

All samples were thawed at room temperature and quantification of TGF-β1 was performed using an ELISA^[9]^ and techniques that have previously been validated for the quantification of TGF-β1 (24). All samples were run in duplicate and all samples were run on the same day. Data were subsequently analyzed using 4PL software^[10]^.

### Modified ACP Preparation Protocol

Based upon the results of the aforementioned methods we sought to evaluate the cellular composition of ACP prepared using a protocol that was modified slightly from that described above. Seventeen dogs owned by students and staff as well as one client-owned dog at the University of Georgia were enrolled, all of whom provided written consent. All dogs were healthy and all were a minimum of 15 kg except for one client-owned toy poodle that weighed under 5 kg. All dogs were between 1 and 10 years of age. No dogs were taking any medications other than nutritional or nutraceutical supplements such as glucosamine and chondroitin.

All dogs had one ACP syringe, that was pre-loaded with 1.5 mls of ACD-A, filled with blood obtained from a jugular vein using a 19-gauge butterfly catheter. No dogs were sedated for blood collection. A half milliliter of blood was placed in a small purple top tube with EDTA for a baseline complete blood count. The ACP syringe was then spun at 1500 RPM for 5 min with the break disengaged. ACP was harvested after this first centrifugation cycle if a minimum of 2 mls of ACP could be obtained. If there were insufficient separation of erythrocytes and plasma to obtain 2 mls of ACP the syringe was centrifuged for an additional 5 min at 1,500 RPM, rather than an additional 10 min as had been done in the prior portion of this study. ACP was then obtained and a sample was placed in EDTA for a CBC. The samples were assessed using a complete blood count and the cellular composition of the ACP and whole blood were then characterized.

### Statistical Analyses

#### ACP Cellular Data

The cellular data including the mean platelet and white blood cell concentrations in the ACP relative to those in the whole blood were summarized. The platelet capture efficiency was calculated by dividing the total number of platelets in each ACP sample by the total number of platelets in the associated whole blood from which the ACP was derived.

#### ACP Activation and TGF-β1 Concentration

A Skillings-Mack test on ranks was performed to evaluate the effect of activation method on the TGF-β1 concentration^[11]^. A Skillings-Mack test was performed because we elected to omit one outlying TGF-β1 concentration of 61,305 pg/ml. This aliquot had not been activated and the range of TGF-β1 concentrations for the other 14 samples that had not been activated was just 2,092–9,647 pg/ml. The concentration of 61,305 pg/ml was 6 times more than the next closest aliquot that had not been activated and was also higher than any other value in the entire study, including all aliquots that had been activated with either calcium chloride or thrombin. Consequently, we believed this data point was erroneous and that exclusion from the analysis was appropriate.

Pair-wise comparisons between activation groups were made using one-tailed Wilcoxon signed rank tests^[9]^. The Pearson product correlations between TGF-β1 and platelet concentrations in the ACP were assessed for all activation treatment groups using an online calculator^[12]^.

#### Characteristics Influencing ACP Platelet and TGF-βΒ1 Content

The amount of platelet concentration and leukocyte reduction relative to the baseline whole blood was compared between samples that were centrifuged just once versus multiple times during the preparation of the ACP using two sample *t*-tests^[13]^. These *t*-tests were performed for samples made in part 1 of this study (*n* = 15 samples), part 2 of this study (*n* = 17 samples), and when data from parts 1 and 2 of this study were pooled (*n* = 32). Similarly, the TGF-β1 concentrations were compared between ACP samples that required only 1 centrifugation during preparation and those that required multiple centrifugations using a *t*-test (*n* = 15). Using the pooled data from all 32 dogs, a multiple logistic regression analysis^[14]^ was used to assess whether dog age, bodyweight, or whole blood hematocrit were predictors of whether one versus multiple centrifugations were needed for ACP preparation. A multiple linear regression analysis^14^ was used to evaluate whether dog age, bodyweight, or whole blood hematocrit were predictors of ACP platelet concentration for all 32 dogs.

## Results

The mean age of dogs in part 1 of this study was 4.2 years (2.5), 5.2 years (2.7) for dogs in part 2 of the study, and 4.8 years (2.6) when data from all 32 dogs were pooled. The mean weight was 29.1 kg (9.7), 25.2 kg (9.5), and 27.0 kg (2.6) for dogs in parts 1, 2, and when the data for all 32 dogs were pooled respectively.

### ACP Cellular Composition

The cellular characteristics of the whole blood and ACP samples from dogs in both parts of this study are shown in Table 1 below.

**Table 1:** Cellular concentrations (×1,000/µL) in the whole blood and ACP.

		**WBC**	**PMN**	**Lym**	**Mon**	**Eos**	**Bas**	**HCT**	**Platelet**
		**Whole Blood**							
Part 1 (*n* = 15)	Mean	10.0	7.0	2.0	0.4	0.6	0.0	50.8	240.9
	Std	3.1	2.2	0.9	0.2	0.5	0.0	2.5	43.7
Part 2 (*n* = 17)	Mean	7.6	5.2	1.7	0.2	0.4	0.0	48.3	235.9
	Std	2.1	1.8	0.6	0.1	0.3	0.0	5.2	47.9
Pooled (*n* = 32)	Mean	8.7	6.1	1.8	0.3	0.5	0.0	49.5	238.3
	Std	2.9	2.1	0.7	0.2	0.4	0.0	4.3	45.3
		**ACP**							
Part 1	Mean	3.1	0.7	1.8	0.2	0.3	0.0	1.4	279.5
	Std	5.1	1.6	2.5	0.3	0.8	0.0	3.8	142.7
Part 2	Mean	2.2	0.3	1.6	0.1	0.1	0.0	0.7	323.8
	Std	1.2	0.3	0.9	0.1	0.1	0.0	1.3	172.5
Pooled	Mean	2.6	0.5	1.7	0.2	0.2	0.0	1.0	303.0
	Std	3.6	1.1	1.8	0.2	0.6	0.0	2.7	158.3

The concentrations are reported as WBC, white blood cells; PMN, polymorphonuclear cells; Lym, lymphocytes; Mon, monocytes; Bas, basophils; HCT, hematocrit.

Additional summary statistics for the ACP including the volume of ACP produced and degree of platelet concentration and leukoreduction are shown in Table 2.

**Table 2:** Summary of platelet concentration, leukoreduction, and platelet capture efficiency of the ACP preparation protocol.

		**Centrifugation Time (mins)**	**# spins**	Volume (ml)*	**PlateletX**	**WBCX**	**Efficiency**
Part 1	Mean	9.7	1.5	5.5	1.2	0.3	21.6
	Std	5.2	0.6	1.3	0.6	0.4	11.5
Part 2	Mean	7.1	1.4	2.4	1.4	0.3	21.1
	Std	2.5	0.5	0.3	0.6	0.1	10.0
Pooled	Mean	8.3	1.5	3.9	1.3	0.3	21.3
	Std	4.1	0.6	1.8	0.6	0.3	10.6

PlateletX = ACP platelet concentration / whole blood platelet concentration. WBCX = ACP WBC concentration / whole blood WBC concentration. Efficiency = (Volume ACP * Platelet concentration) / (Volume whole blood * platelet concentration).

* Thirty milliliters of anti-coagulated whole blood were used in part 1 of this study and 15 mls of whole blood was used in part 2 of this study.

### TGFβ−1 Concentrations

**Figure 1:**
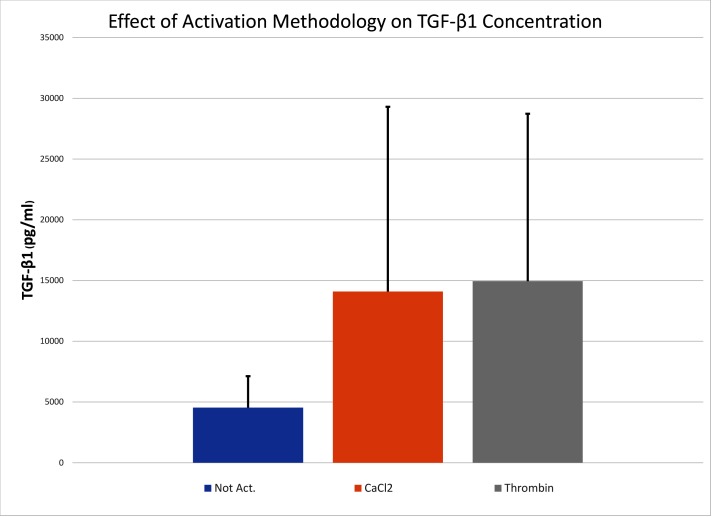
Mean (SD) of the TGF-β1 in ACP that was not activated (Not Act), activated with human gamma thrombin (Thrombin), or activated with calcium chloride (CaCl_2_). The error bars represent SD.

The concentrations of TGF-β1 for each of the 3 activation groups are shown in Figure 1. With that singular data point omitted, the Skillings-Mack test demonstrated that there were significant differences among treatment group (*p* < 0.05). Based upon pairwise comparisons using the Wilcoxon rank-sum test, samples that were activated with either calcium chloride or thrombin had significantly higher TGF-β1 concentrations than samples that were not activated (*p* < 0.05 for both comparisons). There was not a significant difference in TGF-β1 concentrations between samples that were activated with calcium chloride or thrombin (*p* > 0.05).

There was no significant correlation (*p* > 0.05) between the TGF-β1 concentrations in aliquots that were not activated and the platelet concentrations in the ACP. There were significant correlations between the platelet concentrations and the TGF-β1 concentrations in aliquots that were activated with calcium chloride (r = 0.86; *p* < 0.0001) or thrombin (r = 0.66; *p* < 0.01).

### Characteristics Influencing ACP Platelet and TGF-β1 Content

Of the ACP prepared from 15 dogs in part 1 of this study, only one centrifugation was needed to prepare at least 2 mls of ACP (per ACP syringe) for 8 dogs. The mean platelet concentration in the ACP from those 8 dogs was 1.7× (0.4) that of the baseline whole blood. For the 7 dogs that had multiple centrifugations performed to produce ACP, the mean platelet concentration was 0.6× (0.2) that of whole blood. The difference in degree of platelet concentration between ACP samples that were prepared with 1 versus multiple centrifugations was statistically significant (*p* < 0.0001). For part 2 of this study (*n* = 17 dogs), just 1 centrifugation was required in order to prepare at least 2 mls of ACP from 10 dogs while 2 centrifugations were required for ACP preparation from 7 dogs. The mean degree of platelet concentration above that in whole blood was 1.6× (0.6) for samples that were centrifuged just once and 0.9× (0.3) for samples that were centrifuged twice. This difference was statistically significant (*p* < 0.05). When data were pooled for all 32 dogs, the mean platelet concentration above that in the whole blood was 1.7× (0.5) for samples that were prepared with 1 centrifugation and was 0.8× (0.3) for samples that required multiple centrifugations. This difference was significant (*p* < 0.0001). When data from all 32 dogs were pooled, dog body weight, age, and hematocrit were not significant predictors (*p* > 0.05) of whether 1 or more centrifugations were performed when preparing the ACP, nor were these variables significant predictors of the final platelet concentration in the ACP.

For ACP prepared with just 1 centrifugation in part 1 of this study the white blood cell concentration was 0.4× (0.4) that of whole blood. For samples that were centrifuged multiple times the leukocyte concentration was 0.06× (0.07) that in whole blood. This difference was statistically significant (*p* < 0.05). In part 2 of the study the white blood cell concentration was 0.3× (0.1) that of the whole blood for ACP samples that were prepared from 1 centrifugation and the leukocyte concentration was 0.25× (0.2) for ACP samples that were prepared with 2 centrifugations. This difference was not statistically significant (*p* > 0.05). When the data from all 32 dogs were pooled, samples that were prepared with a single centrifugation had a leukocyte concentration of 0.4× (0.3) that of whole blood and samples prepared with multiple centrifugations had a leukocyte concentration of 0.2× (0.2). This difference was statistically significant (*p* < 0.05).

## Discussion

The primary objective of this study was to try and confirm the cellular composition of the ACP samples given the highly variable results in previously published studies (2, 3, 5, 16). The data from this study showed that on average the ACP has a platelet concentration about 1.3× (i.e., 130%) that in comparison to the whole blood and a mean leukocyte concentration that was just 27% of that in the whole blood. Moreover, of the specific leukocyte types, the neutrophils were reduced to below 10% of their concentration in the baseline whole blood. These results are similar to those of all 4 previous studies in that all 4 studies reported ACP to be leukoreduced in comparison to whole blood (2, 3, 5, 16). However, the studies differ in regard to the reported degree of platelet concentration in the ACP with the studies by Cook et al (2015), Bozynski et al (2016), Stief et al (2011), and Carr et al (2015) reporting mean platelet concentrations of 2.5, 2.4, 1.0, and 0.09× that in whole blood (2, 3, 5, 16). The results from this study of 1.3X fall in the middle of these values with the most discrepant result being that showing a platelet concentration of 0.09× (5). The reasons for the discrepancy among studies could include different methods of ACP preparation as specific details regarding preparation protocols were not comprehensively delineated in all studies (2, 3, 5). Such differences could include differences in centrifugation times, centrifugation speeds, number of centrifugation cycles, different methods of anti-coagulation, and different methods of cellular counting. In addition, we speculate that differences in manual collection of the ACP is likely to explain the one study result in which the ACP was essentially devoid of platelets (5). Collection of ACP requires manually aspirating the plasma (i.e., ACP) from the buffy coat and packed red blood cells following centrifugation. If the user only aspirates the superficial layer of plasma, and does not collect the layer of plasma closest to the buffy coat, one is only collecting the platelet-poor plasma. Although the exact reasons for discrepancy among studies is unclear, we believe the results of this current study are accurately representative of ACP preparation using the techniques described herein given that the methodology is specifically detailed, this study included more dogs than any other study, and that results were relatively consistent in both parts of this study.

One of the unexpected findings of this study was that multiple centrifugations would be needed for multiple samples and that significantly different results are clearly obtained for samples that require a single versus multiple centrifugation preparations. None of the previous studies reported using multiple centrifugation cycles (2, 3, 5, 16). We found that 18 of 32 samples required multiple centrifugations to achieve adequate separation. Furthermore, the data demonstrate that samples that were centrifuged twice had significantly decreased platelet and leukocyte concentrations in comparison to samples that were centrifuged only once. This specific finding is not surprising and suggests that with additional centrifugation of either 5 or 10 min (i.e., the “standard” or “modified” protocols used in this study), that platelets are likely driven deeper into the buffy coat layer and become more difficult to extract with the ACP. Assuming that a greater platelet concentration is desirable, this clearly shows that one would like to obtain adequate separation with one centrifugation cycle. There was no evidence that dog age, bodyweight, or whole blood hematocrit predicted whether 1 or more centrifugation cycles would be needed during ACP preparation. However, despite the lack of statistical significance of these potential predictive variables, we are suspicious that improved hydration status may influence whether ACP can be made with one centrifugation cycle. This hypothesis is based upon the observation that the mean hematocrit of all dogs in the study was high (49.5 ± 4.3) and the presumption that a greater plasma volume of the whole blood would potentially contribute to a greater plasma volume post centrifugation. Additional studies could be done to evaluate this hypothesis.

The TGF-β1 concentrations in canine ACP were moderate in aliquots that were not intentionally activated and were significantly higher in aliquots that were activated with either calcium chloride or thrombin. These results are consistent with previous studies of other canine PRPs in which intentional activation with either calcium chloride or thrombin significantly increased either platelet activation, TGF-β1 release, or both in comparison to aliquots that were not activated (8, 10, 23). The lack of a significant difference between the aliquots that were activated with calcium chloride versus thrombin is different than one previous study comparing both these activation protocols and could be attributable to the variability seen in the TGF-β1 concentrations in this study, the use of a conservative non-parametric statistical test, and a potential type II statistical error (23). In any event, the mean values of approximately 14,500 pg/ml in samples that were activated with either calcium chloride or thrombin demonstrate that the ACP prepared in this study does provide substantial growth factor delivery with platelet activation. In turn, these results support a potential mechanism of action by which positive results have been noted with use of ACP in dogs with either experimentally-induced or naturally-occurring disease (1–3). However, it should be acknowledged that there was substantial variability in the TGF-β1 concentrations in the aliquots that were activated with calcium chloride or thrombin. The statistically significant and moderate to strong correlations between the TGF-β1 concentrations and the platelet concentrations suggests that a substantial amount of the variability in TGF-β1 concentrations was attributable to the relatively wide variability in the platelet concentrations in the ACP samples.

There were a few limitations of this study. Most notably, we did not include a comprehensive evaluation of patient hydration status. Consequently, we evaluated the potential correlation between hematocrit and ACP platelet concentration to try and gain some insight as to whether hydration status might influence whether the blood adequately separates after just 1 centrifugation cycle, but a more thorough evaluation of hydration status would be more meaningful. In addition, we assessed results using just 2 different protocols and when ACP was made by 1 person. Results could be different if a protocol with different centrifugation times, speeds, anti-coagulant, or person performing the manual extraction were involved. With that said, the results were quite consistent between the two different protocols we assessed and with a relatively large number of dogs.

In summary, there was variability in the platelet concentration in canine ACP with the majority of samples providing mild platelet concentration while others had a decreased platelet concentration in comparison to that in the baseline whole blood sample. Samples that required only a single centrifugation cycle had increased platelet concentration in comparison to whole blood while samples that did not separate adequately after a single cycle and that required a second centrifugation cycle were significantly more likely to have a reduced concentration of platelets in comparison to whole blood. It is unclear why some samples separated adequately with 1 centrifugation and why others did not. There were also substantial TGF-β1 in all PRP samples albeit with large standard deviations in such concentrations. Pertinently, the TGF-β1 concentrations were moderately to strongly correlated with the platelet concentration in the PRP samples. Hence, the large standard deviations in TGF-β1 concentrations were not really a weakness of the study, but are likely an accurate representation of the variability in platelet concentration that can obtained using this system. Finally, although there was variability in the concentration of the platelets, and accordingly the TGF-β1 concentrations, the ACP was consistently leukoreduced.

## Ethics Statement

This study was approved by the Clinical Research Committee at the University of Georgia. All owners of dogs included in this study provided written consent.

## Author Contributions

Dr. Franklin designed the study, assisted with data collection and data analysis, and wrote the manuscript. Ms. Birdwhistell assisted with data collection, data analysis, and with manuscript composition.

## Conflict of Interest Statement

The study was funded by Arthrex Vet Systems and involved an evaluation of a proprietary system for producing PRP. Dr. Franklin is a consultant for Arthrex Vet Systems.

## Footnotes

[2] Dexdomitor, Zoetis, Florham Park, NJ 

[3] Hospira, Lake Forest, IL

[4] Fenwall ACD-A, Lake Zurich, IL

[5] Element HT5, Heska Corporation, Loveland, CO 

[6] Hettich centrifuge with rotor 1324, Arthrex Vet Systems, Naples, FL 

[7] Calcium chloride, haemonetics, Braintree, MA 

[8] HGT, Haematologic corp, South Burlington, VT

[9] Mouse/Rat/Porcine/Canine TGF-β1 Quantikine ELISA Kit, R&D Systems, Minneapolis, MN

[10] Prism, GraphPad, La Jolla, CA

[11] Stata, College Station, TX

[12] http://www.socscistatistics.com/tests/pearson/default2.aspx

[13] Excel, Microsoft, Redmond, WA 

[14] SAS V 9.3, Cary, NC
